# DNA methylation profiling identifies novel markers of progression in hepatitis B-related chronic liver disease

**DOI:** 10.1186/s13148-016-0218-1

**Published:** 2016-05-05

**Authors:** Müjdat Zeybel, Sezgin Vatansever, Timothy Hardy, Ayşegül Akder Sarı, Fulya Cakalağaoğlu, Arzu Avcı, Gemma Louise Zeybel, Serçin Karahüseyinoğlu, Matthew Bashton, John C. Mathers, Belkıs Ünsal, Jelena Mann

**Affiliations:** Institute of Cellular Medicine, Newcastle University, Newcastle upon Tyne, UK; School of Medicine, Koç University Hospital, Koç University, 4th floor- M-4220. Davutpaşa Caddesi no: 4, 34010 Istanbul, Turkey; Department of Gastroenterology and Hepatology, Katip Çelebi University, Atatürk Eğitim ve Araştırma Hastanesi, Izmir, Turkey; Department of Pathology, Katip Çelebi University, Atatürk Eğitim ve Araştırma Hastanesi, Izmir, Turkey; Bioinformatics Support Unit, Faculty of Medical Sciences, Newcastle University, Newcastle upon Tyne, UK; Human Nutrition Research Centre, Newcastle University, Newcastle upon Tyne, UK

**Keywords:** Epigenetics, Liver fibrosis, DNA methylation, Cirrhosis, Hepatitis B infection

## Abstract

**Background:**

Chronic hepatitis B infection is characterized by hepatic immune and inflammatory response with considerable variation in the rates of progression to cirrhosis. Genetic variants and environmental cues influence predisposition to the development of chronic liver disease; however, it remains unknown if aberrant DNA methylation is associated with fibrosis progression in chronic hepatitis B.

**Results:**

To identify epigenetic marks associated with inflammatory and fibrotic processes of the hepatitis B-induced chronic liver disease, we carried out hepatic genome-wide methylation profiling using Illumina Infinium BeadArrays comparing mild and severe fibrotic disease in a discovery cohort of 29 patients. We obtained 310 differentially methylated regions and selected four loci comprising three genes from the top differentially methylated regions: hypermethylation of *HOXA2* and *HDAC4* along with hypomethylation of *PPP1R18* were significantly linked to severe fibrosis. We replicated the prominent methylation marks in an independent cohort of 102 patients by bisulfite modification and pyrosequencing. The timing and causal relationship of epigenetic modifications with disease severity was further investigated using a cohort of patients with serial biopsies.

**Conclusions:**

Our findings suggest a linkage of widespread epigenetic dysregulation with disease progression in chronic hepatitis B infection. CpG methylation at novel genes sheds light on new molecular pathways, which can be potentially exploited as a biomarker or targeted to attenuate inflammation and fibrosis.

**Electronic supplementary material:**

The online version of this article (doi:10.1186/s13148-016-0218-1) contains supplementary material, which is available to authorized users.

## Background

Hepatitis B is the one of the leading causes of chronic liver disease; 350 million people are chronically infected by the hepatitis B virus worldwide [1]. It has been estimated that hepatitis B infection is responsible for more than 500,000 deaths annually and is the 15th most common cause of death globally [2, 3]. Chronic hepatitis B infection is characterized by immune-mediated liver damage leading to necroinflammation and accumulation of fibrotic tissue. Persistent viral replication and repetitive hepatic injury may ultimately evolve into cirrhosis, together with substantially increased risk of adverse clinical outcomes such as liver failure, portal hypertension and hepatocellular carcinoma [4].

Hepatic inflammation and fibrotic processes comprise complex cellular and molecular interactions. Progression from chronic hepatic inflammation to the fibrotic/cirrhotic stage is underpinned by numerous core pathways, observed in other fibrotic diseases, as well as tissue- or injury-specific pathways that are only activated in particular conditions [5, 6]. Depending on the activity of such mechanisms, progression to fibrosis varies significantly among hepatitis B-infected individuals, as in other liver disorders [4]. To date, several studies have linked genetic and environmental factors to the propensity towards hepatic fibrosis progression. Genetic polymorphisms (patatin-like phospholipase domain-containing protein 3 (PNPLA3), transmembrane 6 superfamily member 2 (TM6SF2), seven-gene signatures) have been particularly associated with the susceptibility of advanced liver disease in non-alcoholic fatty liver disease and chronic hepatitis C infection [7–11]. While the influence of genetic variants in hepatitis B-associated chronic liver disease is less clearly established, a recent study indicated that the rs12979860 polymorphism of interferon-λ is consistently linked to hepatic fibrosis in liver diseases including chronic hepatitis B infection [12].

DNA methylation is the addition of methyl group to a fifth carbon of cytosine residues within a CG dinucleotide, frequently referred to as cytosine-guanine dinucleotide (CpG). This type of DNA methylation is an essential component of epigenetic machinery that regulates the transcriptional state, alongside histone modifications and microRNAs. As a transcriptional regulator, DNA methylation has a considerable impact on the development of common diseases and cancer [13, 14]. Epigenetic mechanisms are dynamically regulated throughout the lifetime and act as an interphase between genetic background and environmental cues. CpG methylation-mediated transcriptional control and epigenetic dysregulation have also been implicated as an important contributing factor in liver diseases including non-alcoholic fatty liver disease and alcoholic liver disease [15, 16]. However, it is not known whether epigenetic signatures influence progression of hepatitis B-related liver disease. We hypothesized that epigenetic signatures can be associated with liver disease progression, and in order to define epigenetic patterns in early and advanced stages of inflammation and fibrosis, we performed an hepatic DNA methylome analysis using liver specimens of chronic hepatitis B patients. Alterations in DNA methylome were further investigated and replicated in the independent cohort and in the cohort of patients with sequential biopsies.

## Results and discussion

**Fig. 1:**
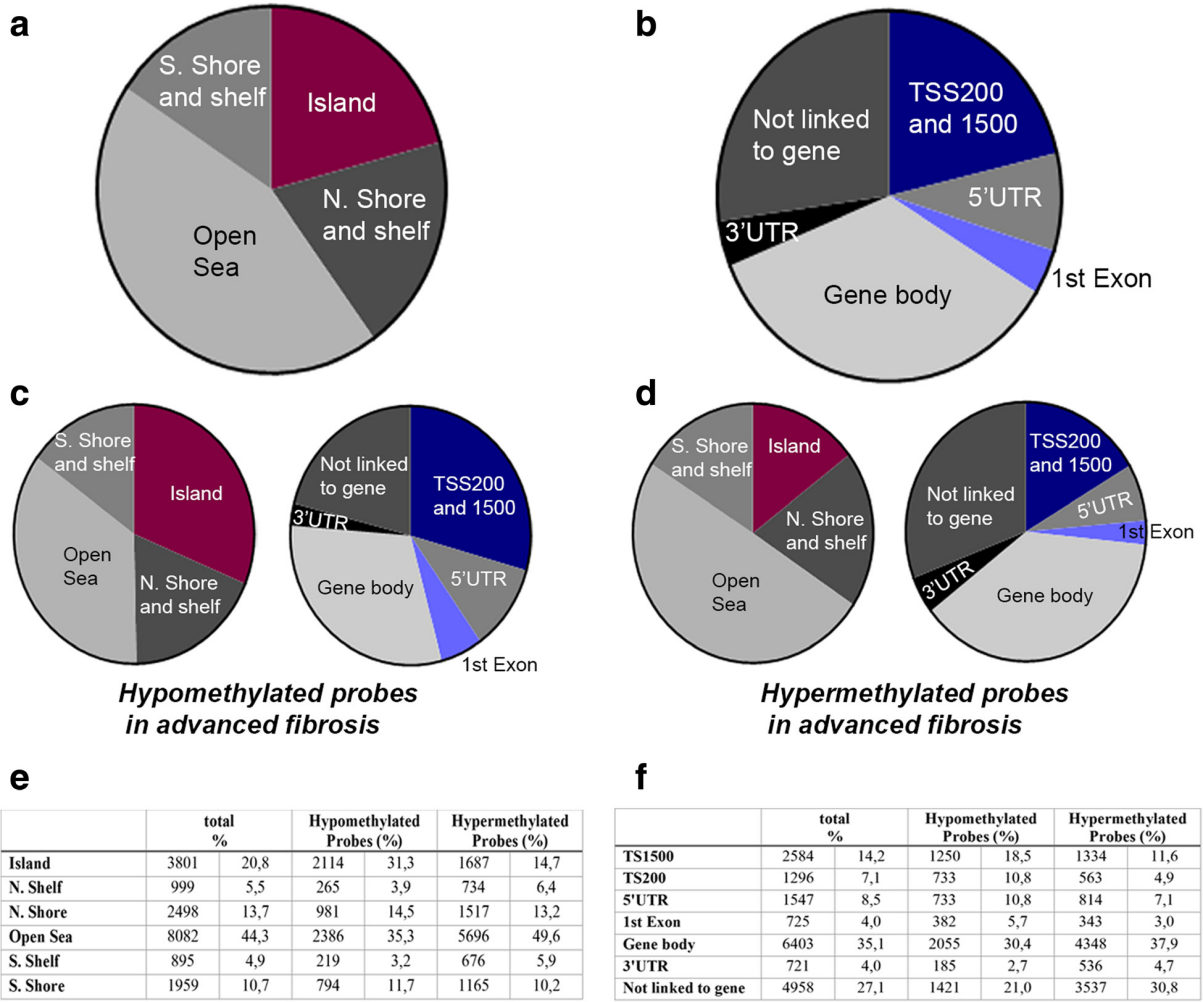
**a** The *pie chart* shows differentially methylated probes in advanced versus mild fibrosis according to UCSC classification of CpG islands: *Island* (CpG island), *N. Shore and shelf* (2-kb upstream of CpG islands and 2–4-kb upstream of CpG islands, flanking regions of shores), *Open Sea*, *S. Shore and shelf* (2-kb downstream of CpG islands and flanking regions of shores). **b** *Pie chart* illustrates probes with methylation differences in advanced versus mild according to functional regions: *TSS200 and 1500* (200- and 1500-bp upstream of transcription start sites; annotated promoters), *5′UTR* (5′ untranslated region), *1st Exon*, *Gene body*, *3′UTR* (3′ untranslated region). **c** *Pie charts* show hypomethylated probes in advanced inflammation and fibrosis in comparison to mild fibrosis according to CpG clusters and functional regions. **d** *Pie charts* show hypermethylated probes in advanced inflammation and fibrosis in comparison to mild fibrosis according to CpG clusters and functional regions. **e**–**f** Number and percentages of differentially methylated probes

To identify DNA methylation alterations in hepatitis B-induced liver disease, we conducted an epigenome-wide mapping using Infinium HumanMethylation450 BeadChip platform. Our discovery cohort included 29 hepatitis B virus (HBV)-infected patients allocated into either a mild fibrosis group (13 patients, fibrosis stage ≤2) or severe fibrosis group (16 patients, fibrosis stage ≥3) (for study design, please see Additional file 1: Figure S1). To minimize the effect of the immune stage of the disease, only hepatitis e antigen-negative patients were included into the discovery cohort. The clinical characteristics, histological activity and staging of the discovery cohort are provided in Table 1. Baseline demographics revealed no significant differences between mild and severe groups. The mean CpG sites detected across the study group was 462,891. We identified a total of 310 differentially methylated regions between the mild and severe groups; 109 of them were significantly hypermethylated, and 201 were hypomethylated in the advanced group. Top-ranked 40 differentially methylated regions are summarized in Additional file 2: Table S1; which includes *HLA-DQA1* (major histocompatibility complex, class II, DQ alpha 1), *TMEM57* (transmembrane protein 57) and *HDAC4* (histone deacetylase 4). Differentially methylated position analysis revealed that 18,234 probes showed significant methylation differences between the two groups; 11,475 loci were associated with higher amounts of methylation and 6759 loci were hypomethylated in the severe fibrosis group. The top 20 hypermethylated and hypomethylated probes associated with disease severity are provided in Additional file 3: Table S2 and Additional file 4: Table S3. Figure 1 illustrates the main characteristics of the breakdown of the CpG sites in relation to transcripts and clusters of CpG sites (CpG islands). Hypomethylated CpG sites in advanced disease were more frequently located within or close proximity to CpG islands (31.3 vs 14.7 %) and transcription start sites (29.3 vs 16.5 %).

**Table 1:** Baseline characteristics of the discovery cohort

	Mild fibrosis (F0-2)	Severe fibrosis (F3-6)	*P* value
	*n* = 16	*n* = 13	
Gender—male^a^	9 (56.2)	8 (61.5)	ns
Age (years)^a^	48.2 ± 14.7	54.2 ± 11.6	ns
Hbe Ag positive^a^	– (0)	– (0)	ns
Anti Hbe Ab positive^a^	16 (100)	13 (100)	–
HBV DNA (log IU/mL)^b^	6.2 ± 5.8	8.5 ± 4.7	ns
Serum ALT (IU/L)^b^	56.5 ± 35.6	106.2 ± 87.1	ns
Serum AST (IU/L)^b^	41.7 ± 29.1	75.1 ± 56.1	0.01
White blood cell count (×10^9^/L)^b^	6.2 ± 1.2	5.6 ± 1.4	ns
Platelet count (×10^9^/L)^b^	218.9 ± 52.1	177.9 ± 64.54	ns
Haemoglobin (g/L)^b^	13.8 ± 0.9	13.6 ± 1.6	ns
Albumin (g/L)^b^	4.4 ± 0.3	4.4 ± 0.4	ns
Prothrombin time (s)^b^	11.8 ± 0.8	12.1 ± 0.9	ns
Direct bilirubin (mg/dL)^b^	0.31 ± 0.1	0.37 ± 0.1	ns
Modified histologic activity index^a^			
0–4	9 (56.25)	2 (15.3)	
5–9	7 (43.75)	9 (69.23)	
10–14	–	2 (15.3)	
15–18	–	–	
Fibrosis^a^			
0	9 (56.25)	–	
1	2 (12.5)	–	
2	5 (31.25)	–	
3	–	4 (30.7)	
4	–	3 (23.0)	
5	–	4 (30.7)	
6	–	2 (15.3)	

^a,b^Data are shown as number and percentage (^a^) or mean and standard deviation (^b^). The histologic activity index is a sum of portal inflammation, confluent necrosis, focal lytic necrosis and periportal or periseptal interface hepatitis scores which was expressed on a scale of 18. Fibrosis stage was assessed on a scale of 0 to 6

**Fig. 2:**
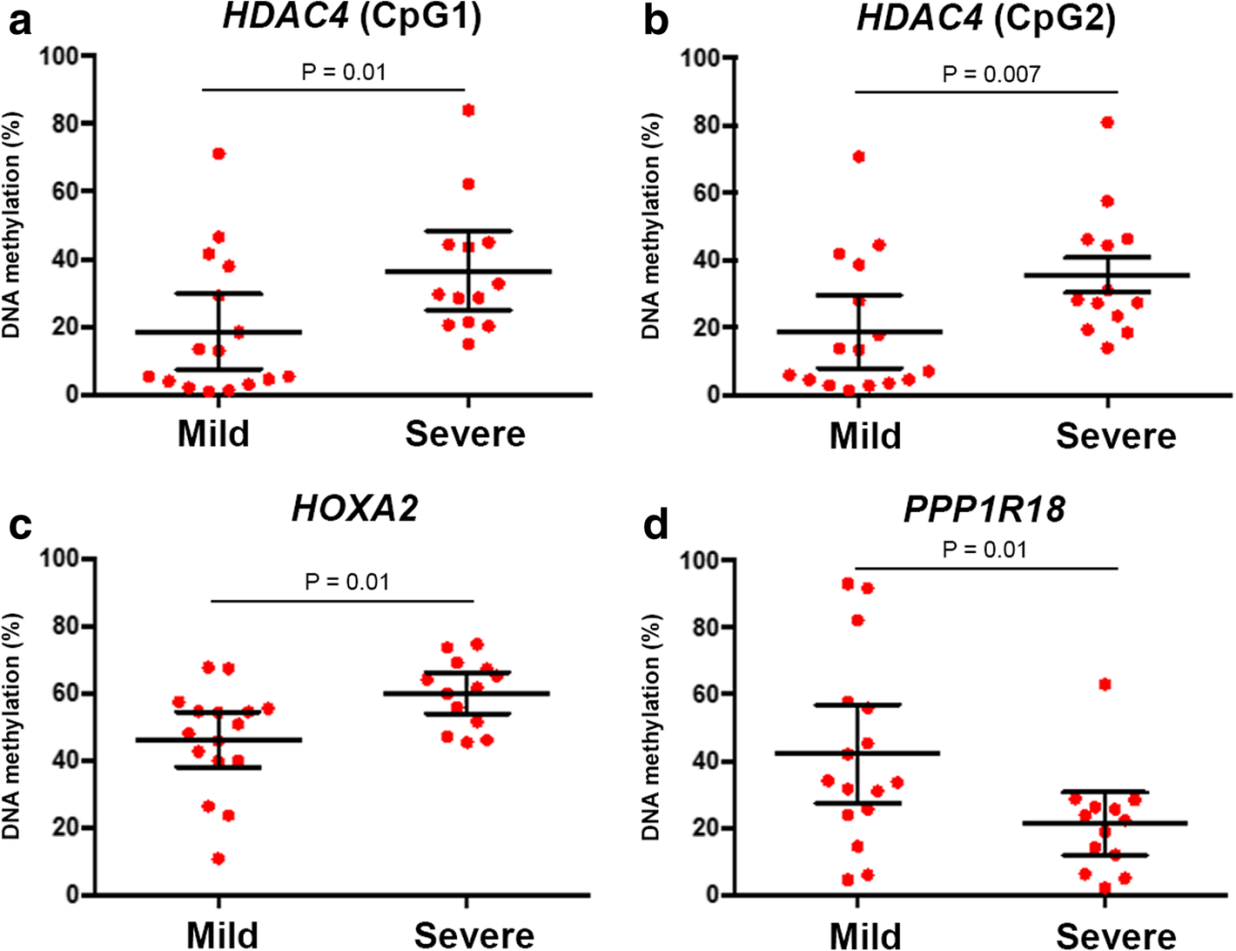
*Plots* were demonstrated as the correlation between the CpG methylation and severity of the liver disease. The analysis compares the hepatic DNA methylation level in the Ishak fibrosis stages of F0-2 with F3-6. *Horizontal bars* represent the means and 95 % confidence interval. Using bisulfite modification and pyrosequencing, hepatic cytosine methylation at four loci was assessed. Changes in HDAC4 (**a**, **b**), HOXA2 (**c**) and PPP1R18 (**d**) CpG methylation are associated with severe inflammation and fibrosis in the discovery cohort

Using bioinformatics assessment and pathway analysis, we identified two individual CpG sites which were strongly associated with the disease progression: cg13985518 (chr7:27143788, *HOXA2* (homeobox A2), CpG site located within 1500 base pairs of transcription start site) and cg20690667 (chr6:30652228, *PPP1R18* (protein phosphatase 1, regulator subunit 18), CpG site positioned within the gene body-1st exon). Further analysis on differentially methylated regions showed that the *HDAC4* gene body (chr2:240241154-240241218) is markedly hypermethylated in the severe group (*P* = 0.002). We further designed pyrosequencing assays to test relevant or nearby CpGs in *HOXA2* and *PPP1R18* genes and two CpGs in the *HDAC4* gene (chr2:240241209-CpG1 and 240241218-CpG2, CpG sites are located in the gene body) by locus-specific DNA methylation analysis in the discovery cohort (Fig. 2). Validation plots of primer sets are provided in Additional file 5: Figure S2; the assays gave satisfactory results with *r*^2^ values over 0.95. We obtained similar DNA methylation changes in *HOXA2*, *PPP1R18* and *HDAC4* genes (*P* values are 0.01, 0.01, 0.01 and 0.007, respectively) as shown in Fig. 2. We also examined the protein levels and localisation of HDAC4 in liver sections of hepatitis B patients (Additional file 6: Figure S3). In the early stages of inflammation and fibrosis, *HDAC* expression was barely detectable in hepatocytes. *HDAC4* positivity was detected in myofibroblast-like cells, inflammatory and biliary cells and hepatocytes adjacent to fibrosis tracts in hepatitis B-induced cirrhosis (Additional file 6: Figure S3).

**Fig. 3:**
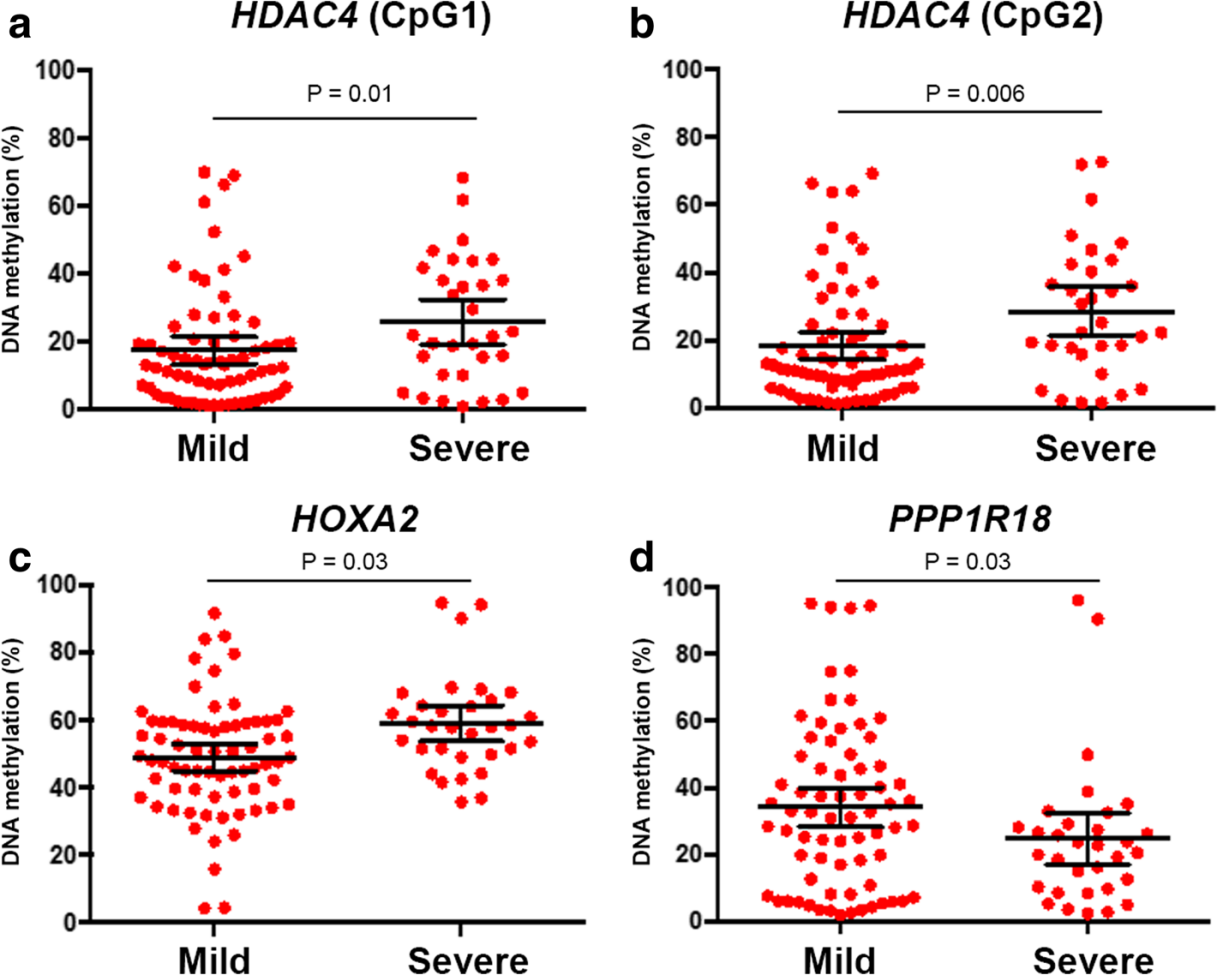
*Plots* were demonstrated as the correlation between the CpG methylation and severity of the liver disease. The analysis compares hepatic DNA methylation level in F0-2 with F3-6 according to Ishak staging. *Horizontal bars* represent the means and 95 % confidence interval. Higher hepatic HDAC4 (**a**, **b**) and HOXA2 (**c**) methylation and lower PPP1R18 (**d**) methylation indicates advanced necroinflammation and fibrosis

We assessed the association of the newly discovered DNA methylation changes in a larger cohort comprising 102 retrospectively recruited patients; in this cohort, 70 patients had mild (Ishak fibrosis stage 0-2) and 32 had severe liver disease (Ishak fibrosis stage 3-6) due to chronic hepatitis B infection. The patient characteristics according to the fibrosis stage are shown in Table 2. Similarly, epigenetic examination was conducted using bisulfite modification and pyrosequencing analysis. As illustrated in Fig. 3, marked increase in *HOXA2* DNA methylation was demonstrated in the severe fibrosis group versus the mild group (chr7:27143806, mean ± SE, 48.8 ± 2.0 vs 58.9 ± 2.5); conversely, significantly lower CpG methylation values were detected in advanced disease at the *PPP1R18* gene (chr6:30652228, mean ± SE, 34.1 ± 2.9 vs 24.5 ± 3.7). Elevated methylation levels in the *HDAC4* gene body associated with progression of necroinflammation and fibrosis were confirmed in previously described two loci (chr2:240241209, 17.5 ± 2.0 vs 25.89 ± 3.27 and chr2:240241218, 18.7 ± 5.0 vs 35.6 ± 5.2).

**Table 2:** Baseline characteristics of the validation cohort

	Mild fibrosis (F0-2)	Severe fibrosis (F3-6)	*P* value
	*n* = 70	*n* = 32	
Gender—male^a^	33 (47.1)	20 (62.5)	ns
Age (years)^a^	45.6 ± 13.7	50.28 ± 11.25	ns
Hbe Ag positive^a^	16	6	ns
Anti Hbe Ab positive^a^	54	26	
HBV DNA (log IU/mL)^b^	7.4 ± 4.3	7.0 ± 2.5	ns
Serum ALT (IU/L)^b^	70.5 ± 48.0	83.0 ± 59.87	ns
Serum AST (IU/L)^b^	45.1 ± 22.5	64.28 ± 47.3	ns
White blood cell count (×10^9^/L)^b^	6.4 ± 1.5	6.3 ± 1.7	ns
Platelet count (×10^9^/L)^b^	207.7 ± 45.6	196.5 ± 42.8	ns
Haemoglobin (g/L)^b^	14.0 ± 1.7	14.5 ± 1.3	ns
Albumin (g/L)^b,c^	4.4 ± 0.3	4.4 ± 0.3	ns
Prothrombin time (s)^b,c^	11.4 ± 1.6	11.5 ± 2.5	ns
Conjugated bilirubin (mg/dL)^b,c^	0.19 ± 0.18	0.26 ± 0.25	ns
Modified histologic activity index^a^			
0–4	47 (67.1)	1 (3.1)	–
5–9	22 (31.4)	25 (78.1)	–
10–14	1 (1.4)	5 (15.6)	–
15–18	–	1 (3.1)	–
Fibrosis^a^			–
0	33 (47.1)	–	–
1	20 (28.5)	–	–
2	17 (24.2)	–	–
3	–	20 (62.5)	–
4	–	5 (15.6)	–
5	–	5 (15.6)	–
6	–	2 (6.2)	–

^a,b^Data are shown as number and percentage (^a^) or mean and standard deviation (^b^). The histologic activity index is a sum of portal inflammation, confluent necrosis, focal lytic necrosis and periportal or periseptal interface hepatitis scores which was expressed on a scale of 18. Fibrosis stage was assessed on a scale of 0 to 6

^c^Information on conjugated bilirubin, prothrombin time and albumin was missing in six, two, and five patients, respectively

**Fig. 4:**
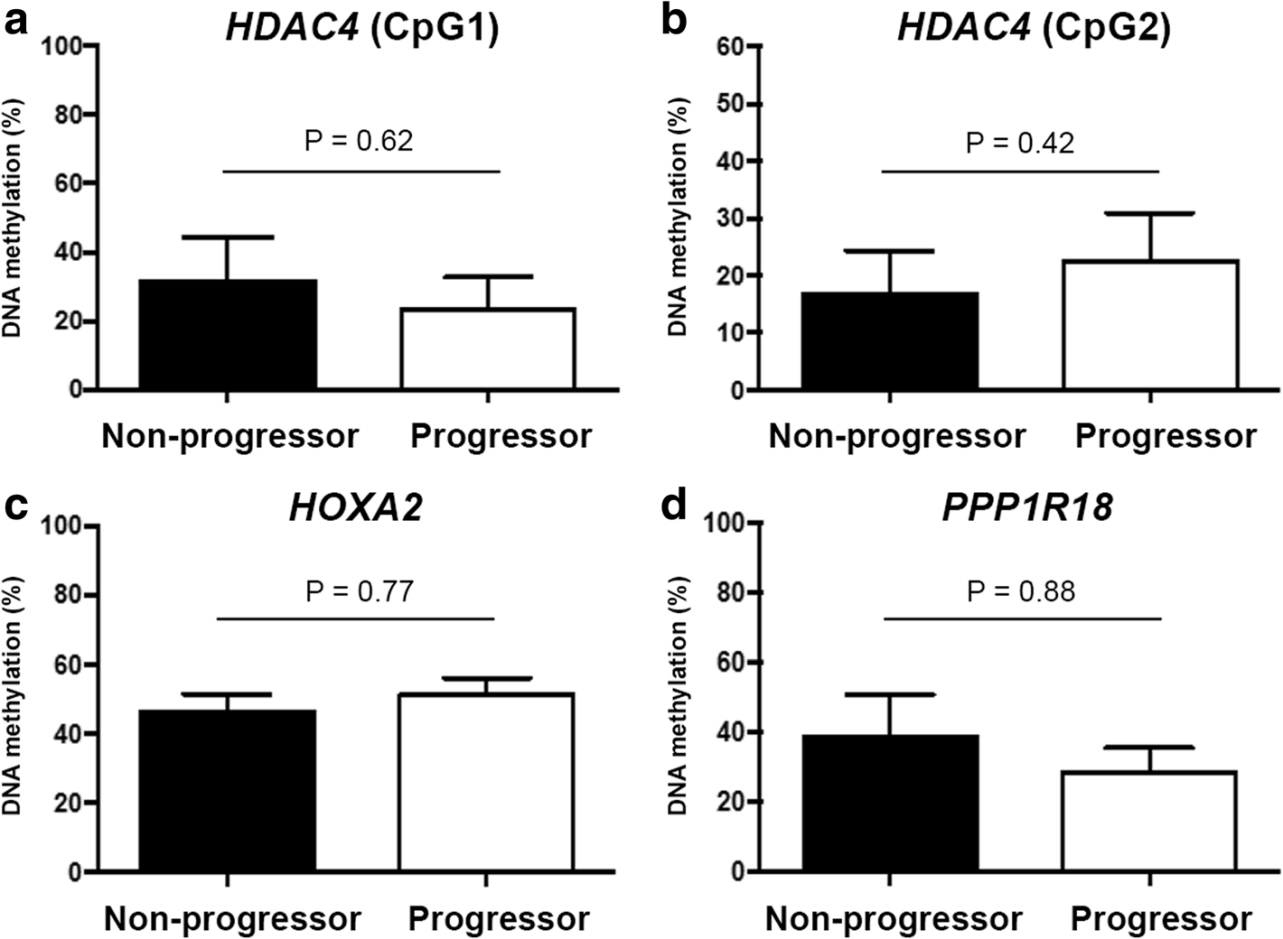
Hepatic DNA methylation levels from initial biopsies of non-progressors (no increase in stage of fibrosis) and progressors (≥1 stage increase in severity of fibrosis). *Boxes* and *bars* represent the means and standard error, respectively. Cytosine methylation at HDAC4 (**a**, **b**), HOXA2 (**c**) and PPP1R18 (**d**) genes did not differ significantly between the two groups

Given the dynamic status of epigenetic modifications, we next considered whether methylation variations in the observed regions developed as a consequence of disease progression. To assess the causality and association of epigenetic imprints, we performed methylation analysis in a cohort of patients with sequential biopsies. Patients were included either into the progressor group (fibrosis stage was increased between two biopsies) or into the non-progressor group (fibrosis stage remained stable) (Additional file 7: Table S4). The comparison of DNA methylation analysis in two groups demonstrated that methylation signatures were not different at initial biopsies (Fig. 4). We further examined the methylation changes using initial and follow-up biopsies. This analysis indicated that only *HOXA2* methylation was increased in the progression of hepatitis B-induced chronic liver disease (Additional file 8: Figure S4).

To our knowledge, this is the first epigenome-wide methylation study comparing mild and severe hepatitis B-related liver disease. Analysis of genome-wide differentially methylated CpG sites and regions in the discovery cohort revealed widespread alterations in the DNA methylome in the progression of chronic liver disease; 310 differentially methylated regions (109 hypermethylated CpG sites and 201 hypomethylated CpG sites) were linked to severe hepatic inflammation and fibrosis. We identified hypermethylation of three loci in *HDAC4* and *HOXA2* genes and a less methylated CpG site in the *PPP1R18* gene in HBV-associated advanced liver fibrosis. Epigenetic differences at four newly identified CpG sites have not previously been linked with chronic liver disease. Observed differences were also confirmed by bisulfite conversion and pyrosequencing in an independent patient cohort. Prior studies on epigenetics of chronic liver disease and hepatocellular carcinoma have noted the importance of DNA methylation in disease development [17, 18]. Furthermore, recent research also suggests that hepatitis B infection induces genome-wide methylation changes in hepatocytes [19, 20]. The current study, while supporting previous findings, also expands our understanding of methylome alterations in histologically proven hepatitis B-induced inflammation and fibrosis.

*HDAC4* is a member of class II histone deacetylases and is located in both nuclear and cytoplasmic compartments of cells. Methylation differences in the *HDAC4* gene have a particular importance, since *HDAC4* executes deacetylation of histone proteins and changes chromatin configuration, which results in transcriptional repression. Given that CpG methylation in the gene body is frequently observed in transcriptionally active genes, higher expression of *HDAC4* might be expected in the severe liver disease group [21]. Indeed, we observed the abundance of *HDAC4* expression in fibrosis tracts of hepatitis B-cirrhosis; conversely, it was significantly less expressed in mild disease. It is likely that the presence of CpG methylation within the *HDAC4* gene may lead to the removal of acetyl groups from histone tails, further repressing transcription. The results of this study are consistent with recent in vitro studies indicating that *HDAC4* plays a key role in myofibroblastic differentiation and fibrogenesis [22]. Furthermore, there is growing evidence that pharmacological inhibition of HDACs by trichostatin A, largazole or valproic acid suppresses fibrogenesis in primary hepatic stellate cells and animal models of liver fibrosis [23–25]. Crosstalk between DNA methylation and histone modifications has been studied extensively [26]. This study raises the possibility that a multifaceted interplay of epigenetic mechanisms may control the progression of chronic liver disease. A potential complementary mechanism for regulation of inflammation and matrix remodelling may be the interaction of genetic variations and epigenetic machineries, e.g. the presence of single nucleotide polymorphisms at the HDAC gene may be a promising tool to predict treatment response in chronic hepatitis C infection [27].

*HOXA2* is part of the *HOXA* transcription factor cluster which has an important role in cell differentiation, embryonic development and cancer. Recent studies suggest that *HOXA* cluster gene expression is under epigenetic control; it is repressed by epigenetic imprints including 5-methylcytosines [28]. Similar epigenetic linkage of the *HOXA* cluster was observed in hepatic malignancies; aberrant methylation of *HOXA2* CpG islands is often associated with cholangiocarcinoma [29]. DNA methylation in promoter regions and CG-rich regions is frequently associated with gene silencing; since we obtained hypermethylation of the *HOXA2* locus within 1500 base pairs of the transcription start site in the current study, this may lead to repression of the *HOXA2* gene in advanced liver disease. Future studies will be needed to characterize a comprehensive function for *HOXA2* in viral-hepatitis-related liver disease. The *PPP1R18* gene (also referred as KIAA1949) encodes a phostensin protein which is localized closely with cytoskeletal proteins such as actin filaments. Phostensin is mainly expressed in lymphocytes, monocytes and macrophages and shown in many tissues, but higher expression is detected in the spleen and other lymphatic tissues [30, 31]. Since lower methylation levels were obtained in the first exon of the *PPP1R18* gene, it is likely that phostensin is up-regulated around inflammatory and fibrotic milieu in correlation with disease severity.

Epigenetic modifications can be dynamically regulated, both in development and disease. In this study, we were not able to determine if the observed epigenetic differences are the cause or the consequence of the disease process. It is likely that our observed patterns of methylation, particularly in CpG-rich gene regulatory regions, can modulate inflammatory and fibrogenic pathways; equally, epigenetic marks could have been acquired subsequently as an outcome of advanced disease. Moreover, contribution of heritable epigenetic factors to liver fibrogenesis has recently been described; therefore, it is also possible that epigenetic signatures can be inherited across generations and modify hepatic disease outcome [32, 33]. Data from the initial biopsies of our follow-up cohort support the idea that DNA methylation variations may be developed with disease progression. Indeed, we observed a modest increase in *HOXA2* methylation in progressors compared to mild reduction in non-progressors. Nevertheless, it is difficult to reach a definite conclusion due to the limited sample size.

It is important to note that this epigenetic study was performed using liver needle biopsy samples that contain several cell types including hepatocytes, myofibroblasts, endothelial cells, macrophages and other inflammatory cells. In line with all other epigenetic modifications, it is likely that some methylated cytosines are cell type-specific, which can impact on the precision of the epigenome-wide studies such as this [34]. Another limitation of the study was the use of moderate sample size in the discovery cohort. We aimed to overcome this issue by using a larger validation cohort to confirm the initial results. Indeed, all four CpG sites were replicated in the validation cohort. It is also worth noting that in our study we employed liver needle biopsy samples, which can represent 1/50,000 of the whole liver. Sampling variability is therefore another issue that has previously been observed in the evaluation of chronic liver disease [35]. We have previously shown that CpG methylation levels are rather consistent across the liver; therefore, sampling error appears to be less of a problem, at least for hepatic DNA methylation [16].

Exploring the epigenetic and cellular basis of chronic liver disease progression is undoubtedly important. There is a growing tendency towards the use of epigenetic modifications as biomarkers of disease in clinical settings. Furthermore, it is also likely that DNA methylation blueprint from the blood or liver may have utility in diagnosing inflammatory or fibrotic status of the liver [36]. A recently published study suggests that the PPARγ promoter hypermethylation in peripheral blood mononuclear cells is associated with severe inflammation and fibrosis in chronic hepatitis B [37]. Our research also suggests that PPARγ promoter hypermethylation of cell-free DNA is a promising biomarker for non-invasive diagnosis of fibrosis in non-alcoholic fatty liver disease [38]. Viral hepatitis-induced chronic liver disease is slowly progressive and a particularly heterogeneous clinical condition. Stratification of patients, and establishment of personalized medicine, needs significant improvement which may be overcome by implementation of DNA methylation markers. Moreover, the highly dynamic nature of epigenetic markers makes them suitable drug targets. Future therapeutic strategies may include the modulation of novel genes involved in inflammation and fibrosis. Further research is required to broaden the mechanistic role of DNA methylation in chronic liver disease.

## Conclusions

In conclusion, we found novel epigenetic signatures associated with hepatic inflammation fibrosis in HBV-related liver disease. These epigenetically modified genes may be linked with liver disease progression and could provide further insights into the pathogenesis of chronic liver disease. Progression-associated CpG sites within *HOXA2*, *PPP1R18* and *HDAC4* genes might have clinical utility as a prognostic marker of HBV-induced chronic liver disease.

## Methods

A total of 131 patients with chronic HBV infection were identified and included retrospectively into the study from a single centre (Katip Çelebi University, Atatürk Eğitim ve Araştırma Hastanesi, Izmir, Turkey). Patients were selected based on the medical records with a positivity for hepatitis B surface antigen (HbsAg) for longer than 6 months and who have undergone percutaneous liver biopsy. Patients that received antiviral therapy in the last 6 months, presence of hepatitis delta infection, hepatitis C infection, non-alcoholic fatty liver disease, drug-induced liver injury, autoimmune liver disease, hereditary hemochromatosis, α1-antitrypsin deficiency or an average alcohol consumption of over 20 g daily for men and over 10 g for women were excluded from the study. Patients with concomitant human immunodeficiency virus (HIV) infection and/or hepatocellular carcinoma were also excluded from the study.

Formalin-fixed paraffin-embedded (FFPE) percutaneous needle liver biopsy specimens were stained with haematoxylin and eosin and picrosirius red/Masson’s trichrome (data not shown). Hepatic inflammatory activity and the fibrosis stage were assessed according to the Ishak system by expert pathologists [39]. The following demographic and clinical laboratory parameters were obtained from medical records: age, sex, aspartate aminotransferase, alanine aminotransferase, conjugated bilirubin, albumin, prothrombin time, complete blood count, HbeAg, anti-Hbe Ab and circulating HBV DNA levels. Genomic DNA was extracted from FFPE needle biopsy samples by QIAamp DNA microkit (Qiagen) and QIA DNA FFPE tissue kit (Qiagen) as recommended by the manufacturer and stored at −80 °C. DNA quality and quantity was assessed by Nanodrop 1000 (Thermo Scientific). DNA samples were hybridized on the Infinium 450K BeadChip (Illumina, San Diego, USA) to evaluate genome-wide DNA methylation. 1 μg DNA was used for bisulfite conversion and repaired by Infinium HD FFPE restore kit (Illumina, San Diego, USA). Amplification and hybridization of samples were performed according to the manufacturer’s protocol. Infinium HumanMethylation450 BeadChip platform data was analysed and processed using Bioconductor minfi package (www.bioconductor.org) [40]. It employs Storey FDR correction [41] where differentially methylated regions are detected using a permutation test, which means the *P* values returned are inherently robust to false discovery. Additionally, during analysis, we used 10,000 permutations, which give a very robust answer, given that the standard default for such analysis is 1000. CpG methylation values for each of the samples were obtained as *β* values. Quality control was implemented, and any *β* scores with a *P* value greater than 0.05 in 12.5 % of the samples were excluded. For correction of systematic differences between type I and type II probes, subset-quantile within array normalization (SWAN) was performed. Probes targeting single nucleotide polymorphisms and probes on sex chromosomes were removed from the downstream analysis. One specimen from a severe group was excluded from further analysis due to failing to provide optimal scores in quality checks. Bump hunter was used to detect differentially methylated regions and Bioconductor minfi package was employed to identify differentially methylated loci [42, 43]. A *P* value was calculated for the probes, and *P* value over 0.05 was considered as not significant.

Validation of genome-wide methylation analysis was performed by bisulfite modification and quantitative pyrosequencing. DNA methylation values from HumanMethylation450 BeadChip array probes were compared with the data obtained from conventional quantitative pyrosequencing. For validation and verification experiments, bisulfite conversion of 1 μg DNA was performed by EZ DNA Methylation GoldTM kit (Zymo Research, Cambridge Bioscience, UK). The conversion protocol was performed as follows: 98 °C for 10 min, 64 °C for 2.5 h and 4 °C holding step. Bisulfite-treated DNA was transferred to spin columns, desulphonated and eluted. Bisulfite-modified DNA was amplified by PCR with the assays and primers designed in our laboratory using Pyromark Assay Design Software SW2.0 (Qiagen, Mainz, Germany). PCR reaction was carried out using 12.5 μL Hot star Taq Master Mix (Qiagen), 5–8 pmol of forward and reverse primers (one of each primer sets were biotin-labelled) in a 25-μL volume. Forward, reverse and pyrosequencing primers were as follows: AATGGGTGATTTATGTTAGAAGAT, CTTTACCAAATTCCTACCTAAAAA, ATATTGGTATTAGGTTTAAG for *HDAC4*; GGGTTTTGTTGTGGGAAATAGTA, CTCAACCTCCTCACCTCTTTCTAA, CCTCTAACACTATCCAAAAT for *HOXA2*; and GGGTGGTGGTAGGAATTAATAAGA, CAACCTCTCCAACCACAATTAATA, GGGATTTTTTGGTAAGGTA for *PPP1R18*.

Amplification was performed according to the following protocol: 95 °C for 15 min, 50 cycles of 95 °C for 15 s, annealing temperature of 55–60 °C for 30 s, 72 °C for 15 s, followed by 72 °C for 5 min. Biotin-labelled PCR products were captured by Streptavidin Sepharose beads (GE Healthcare, UK) and converted to single-stranded DNA by Vacuum Prep Tool (Qiagen). Sequencing primers were used to anneal single-stranded PCR product at 80 °C for 2 min. Pyrosequencing was performed using Pyromark 96 MD system (Qiagen), and CpG methylation at each site was analysed by Pyro Q CG software (Qiagen). Mean methylation levels from four assays and methylation array were considered as concordant as absolute methylation differences were less than 10 %.

Immunohistochemistry for histone deacetylase 4 was performed on FFPE sections. Slides were dewaxed, rehydrated with alcohol and hydrogen peroxide was used to block endogenous peroxidase activity. Antigen retrieval was performed using citrate buffer (15-M103, Bio-Optica, Italy) in a microwave for 15 min. Sections were incubated with primary antibody (anti-HDAC4 ab184983, Abcam) at 37 °C for 2 h. Slides were washed in TBS-T and incubated with secondary antibody (biotinylated goat anti-polyvalent). After TBS-T washing, sections were incubated with streptavidin biotin peroxidase at room temperature for 10 min. HDAC positivity was shown by diaminobenzidine tetrahydrochloride (DAB). Slides were counterstained by haematoxylin.

### Statistical analysis

Statistical analysis and graph construction were performed using SPSS software (IBM, USA) and GraphPad Prism Software (GraphPad, version 6.0). Results are shown as the mean value ± confidence interval graphically. Means were compared via nonparametric tests (Mann-Whitney *U*) to define differences for not normally distributed data. Pearson’s chi-square tests or Fisher’s exact test was used for categorical variables. A *P* value of less than 0.05 was considered statistically significant.

The study was performed in accordance to the Conference on Harmonization Guidelines for Good Clinical Practice and ethical guidelines of the Declaration of Helsinki. Ethical approval for the study protocol was granted by the Institutional Ethical Committee and Review Board (Atatürk Eğitim ve Araştırma Hastanesi, Izmir).

## Additional files

Additional file 1: Figure S1. Flow chart demonstrating patients who underwent liver biopsy procedure and included into the study. (DOCX 43 kb) Additional file 2: Table S1. Summary of differentially methylated regions. (DOCX 16 kb) Additional file 3: Table S2. Hypermethylated probes in severe inflammation and fibrosis. (DOCX 18 kb) Additional file 4: Table S3. Hypomethylated probes in severe inflammation and fibrosis. (DOCX 16 kb) Additional file 5: Figure S2. Validation of the bisulfite modification and pyrosequencing assays for relevant loci in *HDAC4*, *HOXA2* and *PPP1R18* genes. Plots comparing 0, 25, 50, 75 and 100 % of methylated DNA with obtained methylation results are shown; only assays with an acceptable performance (*r* ^2^ values over 0.95) were used in this study. (TIF 149 kb) Additional file 6: Figure S3. Histone deacetylase 4 immunohistochemistry in mild and severe disease: *HDAC4* is highly expressed in advanced inflammation and fibrosis. Representative ×200 pictures of *HDAC4* immunohistochemistry from five mild (fibrosis stage ≤1) and five severe (fibrosis stage ≥4) chronic liver disease due to hepatitis B infection. DAB-positive staining pattern was detected in myofibroblasts, inflammatory cells and hepatocytes in or near to fibrosis tracts. (TIF 1747 kb) Additional file 7: Table S4. Demographic, clinical and laboratory characteristics of the non-progressors and progressors in serial biopsies. (DOCX 18 kb) Additional file 8: Figure S4. Change in hepatic DNA methylation levels between follow-up and initial biopsies of non-progressors (no increase in stage of fibrosis) and progressors (≥1 stage increase in severity of fibrosis). *Boxes* and *bars* represent the means and standard error of cytosine methylation at HDAC4 (**a**, **b**), HOXA2 (**c**) and PPP1R18 (**d**) genes. (TIF 4146 kb)

## Abbreviations

CpG: cytosine-guanine dinucleotide
HBV: hepatitis B virus
HDAC4: histone deacetylase 4
HIV: human immunodeficiency virus
HLA-DQA1: major histocompatibility complex, class II, DQ alpha 1
HOXA2: homeobox A2
PNPLA3: patatin-like phospholipase domain-containing protein 3
PPP1R18: protein phosphatase 1, regulator subunit 18
TM6SF2: transmembrane 6 superfamily member 2
TMEM57: transmembrane protein 57
