# Supplementary figures and images for: DNA methylation profiling identifies novel markers of progression in hepatitis B-related chronic liver disease
Source: Clin Epigenetics. 2016 May 5;8:48. doi: 10.1186/s13148-016-0218-1 (PMC4857425; doi:10.1186/s13148-016-0218-1)

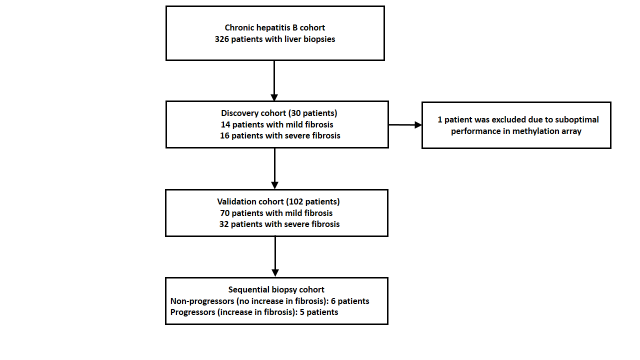

Supplement: Additional file 1: Figure S1. — Flow chart demonstrating patients who underwent liver biopsy procedure and included into the study. (DOCX 43 kb) [file 13148_2016_218_MOESM1_ESM.docx]

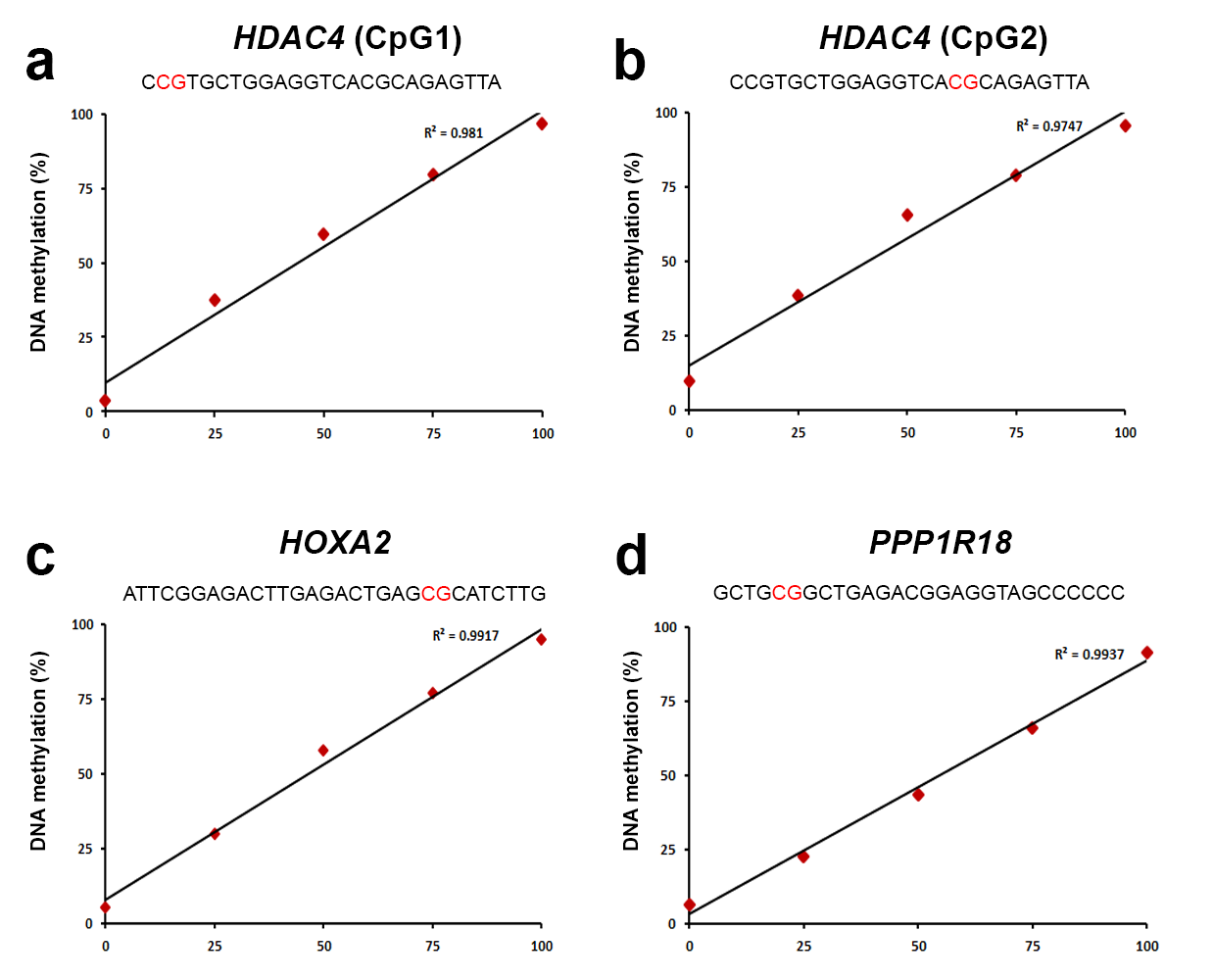

Supplement: Additional file 5: Figure S2. — Validation of the bisulfite modification and pyrosequencing assays for relevant loci in HDAC4, HOXA2 and PPP1R18 genes. Plots comparing 0, 25, 50, 75 and 100 % of methylated DNA with obtained methylation results are shown; only assays with an acceptable performance (r 2 values over 0.95) were used in this study. (TIF 149 kb) [file 13148_2016_218_MOESM5_ESM.tif]

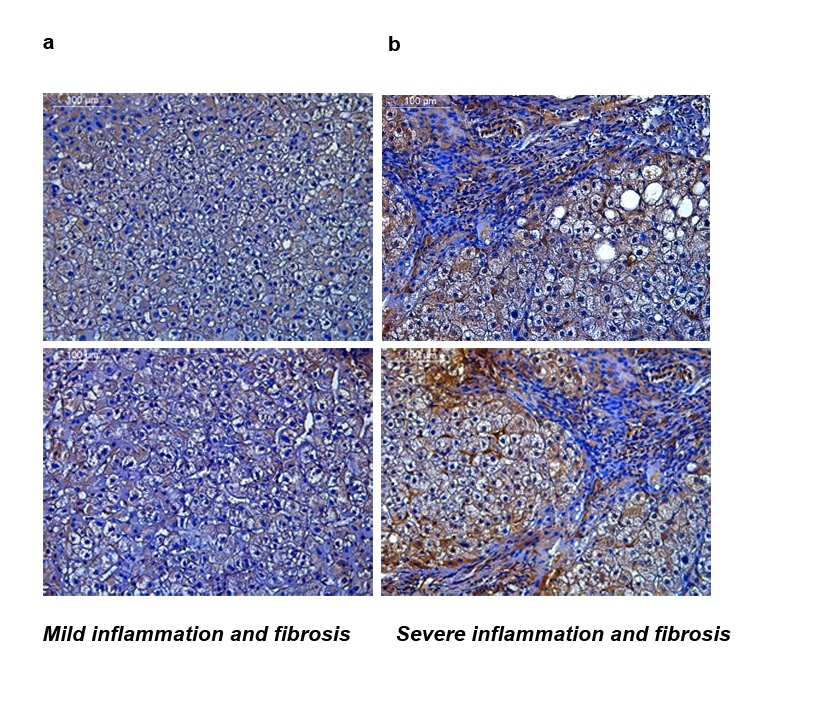

Supplement: Additional file 6: Figure S3. — Histone deacetylase 4 immunohistochemistry in mild and severe disease: HDAC4 is highly expressed in advanced inflammation and fibrosis. Representative ×200 pictures of HDAC4 immunohistochemistry from five mild (fibrosis stage ≤1) and five severe (fibrosis stage ≥4) chronic liver disease due to hepatitis B infection. DAB-positive staining pattern was detected in myofibroblasts, inflammatory cells and hepatocytes in or near to fibrosis tracts. (TIF 1747 kb) [file 13148_2016_218_MOESM6_ESM.tif]

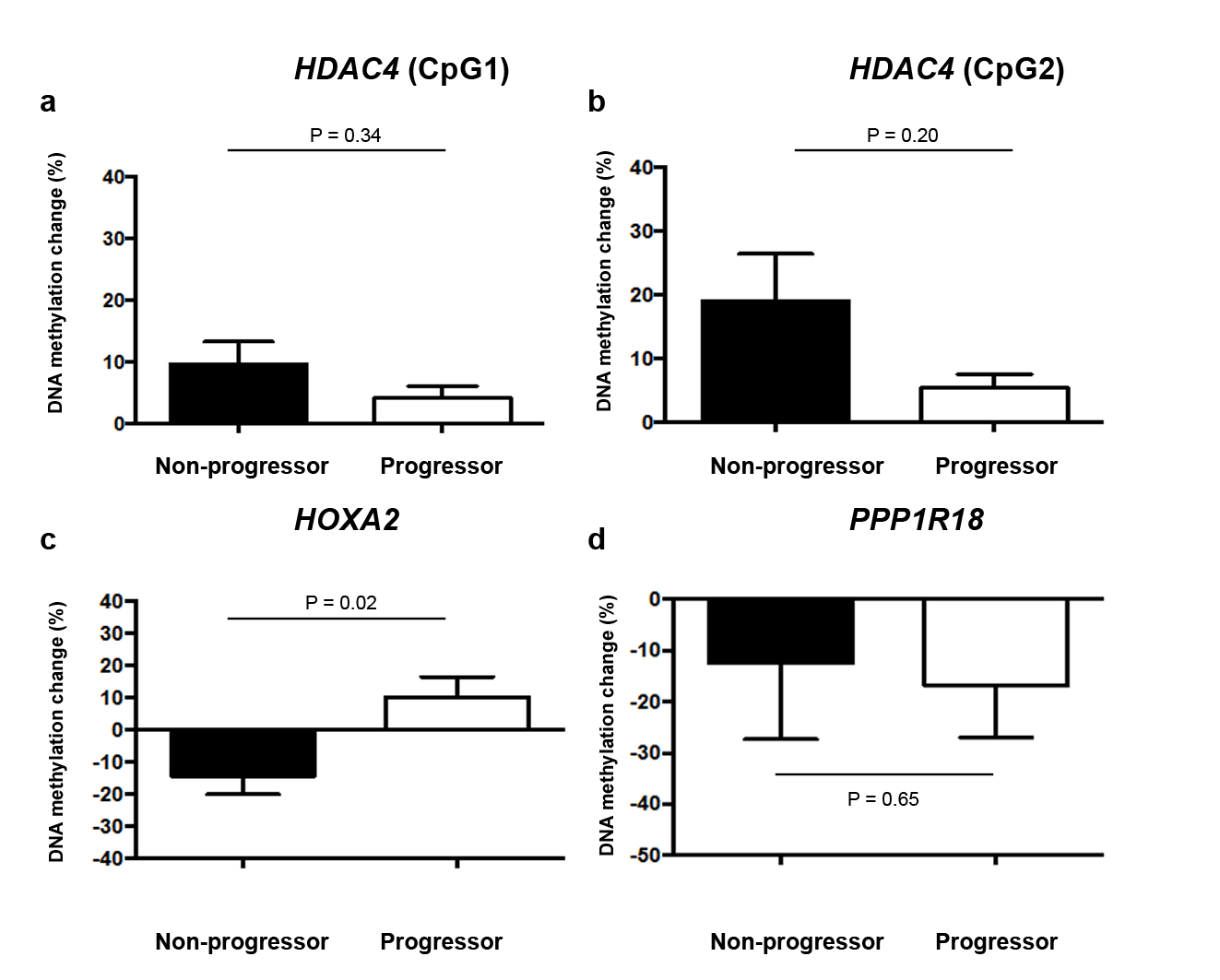

Supplement: Additional file 8: Figure S4. — Change in hepatic DNA methylation levels between follow-up and initial biopsies of non-progressors (no increase in stage of fibrosis) and progressors (≥1 stage increase in severity of fibrosis). Boxes and bars represent the means and standard error of cytosine methylation at HDAC4 (a, b), HOXA2 (c) and PPP1R18 (d) genes. (TIF 4146 kb) [file 13148_2016_218_MOESM8_ESM.tif]
