# Supplementary material for: DNA methylation profiling identifies novel markers of progression in hepatitis B-related chronic liver disease
Source: Clin Epigenetics. 2016 May 5;8:48. doi: 10.1186/s13148-016-0218-1 (PMC4857425; doi:10.1186/s13148-016-0218-1)
Supplement: Additional file 2: Table S1. — Summary of differentially methylated regions. (DOCX 16 kb) [file 13148_2016_218_MOESM2_ESM.docx]

**Supplementary Table 1.**

*Summary of differentially methylated regions*

| Chromosome | Start | Β Value | Cluster | P | Genes |
| --- | --- | --- | --- | --- | --- |
| chr2 | 241896910 | -0,39092283 | 124731 | 0,0001 | uncharacterized LOC200772;rootletin-like |
| chr15 | 65272560 | 0,360665607 | 70680 | 0,0001 | spastic paraplegia 21 (autosomal recessive, Mast syndrome) |
| chr11 | 96076288 | 0,344953824 | 41116 | 0,0001 | mastermind-like 2 (Drosophila) |
| chr1 | 25825780 | -0,336178749 | 5430 | 0,0001 | transmembrane protein 57 |
| chr3 | 127056955 | 0,147760849 | 142492 | 0,0002 | uncharacterized LOC101927123 |
| chr5 | 112073426 | -0,134408476 | 161970 | 0,0002 | adenomatous polyposis coli |
| chr8 | 22266134 | -0,323687683 | 195530 | 0,0002 | solute carrier family 39 (zinc transporter), member 14 |
| chr6 | 29894141 | 0,151372891 | 170821 | 0,0005 | HLA complex group 4B (non-protein coding) |
| chr11 | 123048161 | 0,311584691 | 42937 | 0,0003 | CXADR-like membrane protein |
| chr9 | 82286351 | 0,309302069 | 204861 | 0,0003 | transducin-like enhancer of split 4 (E(sp1) homolog, Drosophila) |
| chr6 | 32609181 | 0,168269097 | 171888 | 0,0008 | HLA class II histocompatibility antigen, DQ alpha 1 chain-like, DQ alpha 1 |
| chr10 | 102279690 | 0,116323514 | 28175 | 0,0009 | NADH dehydrogenase (ubiquinone) 1 beta subcomplex, 8 |
| chr17 | 46682390 | 0,138365273 | 90422 | 0,0011 | HOXB cluster antisense RNA 3 |
| chr1 | 235292369 | -0,288058987 | 20236 | 0,0005 |  |
| chr1 | 151345123 | 0,113455478 | 13230 | 0,0010 | selenium binding protein 1 |
| chr20 | 13200931 | 0,113064541 | 125907 | 0,0010 |  |
| chr11 | 2293201 | 0,161678731 | 32879 | 0,0012 |  |
| chr4 | 140952109 | -0,283368052 | 154021 | 0,0006 | mastermind-like 3 (Drosophila) |
| chr6 | 32607224 | 0,159607446 | 171887 | 0,0014 | HLA class II histocompatibility antigen, DQ alpha 1 chain-like; DQ alpha 1 |
| chr4 | 188916865 | 0,132577067 | 156257 | 0,0015 |  |
| chr19 | 42806201 | -0,130194529 | 105130 | 0,0017 | platelet-activating factor acetylhydrolase 1b, catalytic subunit 3 (29kDa) |
| chr1 | 62499140 | 0,278162573 | 9657 | 0,0007 | InaD-like (Drosophila) |
| chr5 | 137610127 | -0,12883802 | 163491 | 0,0018 | GDNF family receptor alpha 3 |
| chr8 | 128750586 | -0,128351169 | 201181 | 0,0019 | v-myc avian myelocytomatosis viral oncogene homolog |
| chr3 | 46538742 | 0,127827318 | 137965 | 0,0019 | receptor (chemosensory) transporter protein 3 |
| chr5 | 179634671 | 0,153179568 | 167488 | 0,0021 | RasGEF domain family, member 1C |
| chr19 | 49340489 | 0,151442729 | 106359 | 0,0023 | pleckstrin homology domain containing, family A member 4 |
| chr2 | 44065550 | 0,117796372 | 112091 | 0,0031 | ATP-binding cassette, sub-family G (WHITE), member 5 |
| chr2 | 216877750 | -0,116169686 | 121753 | 0,0033 | melanoregulin;peroxisomal trans-2-enoyl-CoA reductase |
| chr7 | 27170394 | 0,114306193 | 183795 | 0,0035 | HOXA cluster antisense RNA 2;HOXA cluster antisense RNA 3;homeobox A3;homeobox A4 |
| chr11 | 57192251 | -0,150134071 | 36667 | 0,0025 | solute carrier family 43, member 3 |
| chr3 | 112929830 | 0,15013329 | 141442 | 0,0025 |  |
| chr1 | 200842756 | 0,148770774 | 16869 | 0,0027 | G protein-coupled receptor 25 |
| chr2 | 240241154 | 0,148599106 | 124369 | 0,0028 | histone deacetylase 4 |
| chr19 | 45737610 | 0,11161731 | 105527 | 0,0038 | MAP/microtubule affinity-regulating kinase 4 |
| chr16 | 88902276 | 0,266470692 | 83468 | 0,0010 | galactosamine (N-acetyl)-6-sulfate sulfatase |
| chr17 | 9019086 | -0,146969647 | 86060 | 0,0031 | netrin 1 |
| chr11 | 66624256 | -0,145787042 | 38553 | 0,0033 | pyruvate carboxylase;leucine rich repeat and fibronectin type III domain containing 4 |
| chr6 | 100895050 | -0,143279127 | 175840 | 0,0039 | single-minded family bHLH transcription factor 1 |
| chr11 | 77122839 | -0,141873855 | 40206 | 0,0043 | p21 protein (Cdc42/Rac)-activated kinase 1 |
