# Supplementary material for: DNA methylation profiling identifies novel markers of progression in hepatitis B-related chronic liver disease
Source: Clin Epigenetics. 2016 May 5;8:48. doi: 10.1186/s13148-016-0218-1 (PMC4857425; doi:10.1186/s13148-016-0218-1)
Supplement: Additional file 3: Table S2. — Hypermethylated probes in severe inflammation and fibrosis. (DOCX 18 kb) [file 13148_2016_218_MOESM3_ESM.docx]

**Supplementary Table 2.**

*Hypermethylated probes in severe inflammation and fibrosis*

| Probe ID | Methylation level in advanced cohort | Methylation level in mild cohort | Delta- change between two groups | P | Chromosome:  position | Relation to Island | UCSC RefGene | RefGene  Group | DMR |
| --- | --- | --- | --- | --- | --- | --- | --- | --- | --- |
| cg05779458 | 0,89 | 0,79 | 0,104 | 1,51505E-07 | chr2:82756573 | OpenSea |  |  |  |
| cg12309703 | 0,84 | 0,72 | 0,115 | 2,05098E-06 | chr2:218621258 | N_Shore | DIRC3 | Body |  |
| cg12590430 | 0,77 | 0,61 | 0,157 | 3,11883E-06 | chr7:38468944 | OpenSea | AMPH | Body |  |
| cg16231394 | 0,82 | 0,75 | 0,074 | 1,38209E-05 | chr5:91137099 | OpenSea |  |  |  |
| cg00594841 | 0,84 | 0,76 | 0,076 | 2,10804E-05 | chr2:196622692 | OpenSea | DNAH7 | Body |  |
| cg18990874 | 0,78 | 0,65 | 0,124 | 2,94179E-05 | chr13:77502558 | OpenSea | BTF3L1 | TSS200 |  |
| cg08533934 | 0,86 | 0,79 | 0,064 | 3,3135E-05 | chr20:59540271 | N_Shelf |  |  |  |
| cg05869585 | 0,91 | 0,87 | 0,043 | 3,34159E-05 | chr16:8890233 | N_Shore | TMEM186  PMM2 | Body;  TSS1500 |  |
| cg26479028 | 0,49 | 0,34 | 0,154 | 3,58636E-05 | chr16:82068702 | OpenSea | HSD17B2 | TSS200 |  |
| cg19243330 | 0,61 | 0,47 | 0,132 | 3,84065E-05 | chr12:53552274 | OpenSea | CSAD | 3'UTR |  |
| cg06616221 | 0,92 | 0,80 | 0,116 | 4,23044E-05 | chr15:48071501 | OpenSea |  |  |  |
| cg08549390 | 0,81 | 0,65 | 0,160 | 4,27353E-05 | chr7:25702709 | OpenSea |  |  |  |
| cg16792706 | 0,93 | 0,86 | 0,069 | 4,78223E-05 | chr15:77908222 | S_Shore | LINGO1 | Body |  |
| cg09771054 | 0,95 | 0,89 | 0,056 | 4,93975E-05 | chr12:130918514 | OpenSea | RIMBP2 | Body |  |
| cg13985518 | 0,53 | 0,38 | 0,154 | 5,51725E-05 | chr7:27143788 | S_Shore | HOXA2 | TSS1500 | CDMR |
| cg00871124 | 0,82 | 0,76 | 0,062 | 5,92143E-05 | chr7:6563852 | N_Shelf | GRID2IP | Body |  |
| cg04698472 | 0,67 | 0,53 | 0,137 | 6,19917E-05 | chr19:52000825 | OpenSea | SIGLEC12 | Body |  |
| cg15448394 | 0,92 | 0,89 | 0,032 | 6,35625E-05 | chr3:57432946 | OpenSea | DNAH12 | Body |  |
| cg19063563 | 0,73 | 0,57 | 0,156 | 6,89005E-05 | chr6:8079840 | OpenSea | EEF1E1 | Body;3'UTR |  |
| cg18223430 | 0,42 | 0,30 | 0,124 | 6,9668E-05 | chr12:12223841 | OpenSea | BCL2L14 | TSS200 |  |
