# Supplementary material for: DNA methylation profiling identifies novel markers of progression in hepatitis B-related chronic liver disease
Source: Clin Epigenetics. 2016 May 5;8:48. doi: 10.1186/s13148-016-0218-1 (PMC4857425; doi:10.1186/s13148-016-0218-1)
Supplement: Additional file 4: Table S3. — Hypomethylated probes in severe inflammation and fibrosis. (DOCX 16 kb) [file 13148_2016_218_MOESM4_ESM.docx]

**Supplementary Table 3.**

*Hypomethylated probes in severe inflammation and fibrosis*

| Probe ID | Methylation level in advanced cohort | Methylation level in mild cohort | Delta- change between two groups | P | Chromosome:  position | Relation to Island | UCSC RefGene | RefGene  Group | DMR |
| --- | --- | --- | --- | --- | --- | --- | --- | --- | --- |
| cg02172819 | 0,51 | 0,61 | -0,102 | 1,12374E-06 | chr10:79261503 | OpenSea | KCNMA1 | Body |  |
| cg06795216 | 0,64 | 0,76 | -0,125 | 8,94249E-06 | chr13:44596456 | OpenSea | LOC121838 | TSS200 |  |
| cg03013422 | 0,11 | 0,24 | -0,128 | 3,16079E-05 | chr12:125550350 | S_Shore | AACS | Body |  |
| cg01087991 | 0,41 | 0,47 | -0,062 | 4,54689E-05 | chr4:110655284 | S_Shelf |  |  |  |
| cg20690667 | 0,33 | 0,51 | -0,175 | 5,74762E-05 | chr6:30652228 | N_Shelf | KIAA1949 | Body;  1stExon | RDMR |
| cg06193597 | 0,38 | 0,77 | -0,391 | 5,77664E-05 | chr2:241896910 | OpenSea |  |  |  |
| cg23866412 | 0,10 | 0,14 | -0,043 | 7,43703E-05 | chr19:18337365 | Island | PDE4C | TSS200;  Body |  |
| cg26269802 | 0,15 | 0,22 | -0,073 | 7,52728E-05 | chr19:8642316 | OpenSea | MYO1F | TSS200 |  |
| cg02936872 | 0,06 | 0,13 | -0,073 | 9,89253E-05 | chr9:99801550 | Island | CTSL2 | TSS200 |  |
| cg25217583 | 0,72 | 0,83 | -0,106 | 0,000104083 | chr1:50574383 | OpenSea | ELAVL4 | Body;TSS1500 |  |
| cg03941709 | 0,05 | 0,07 | -0,022 | 0,000113436 | chr7:87505496 | Island | SLC25A40;  DBF4 | 5'UTR;1stExon;  TSS200 |  |
| cg09892671 | 0,82 | 0,89 | -0,069 | 0,000113961 | chr1:82951578 | OpenSea |  |  |  |
| cg09501372 | 0,19 | 0,30 | -0,107 | 0,000120525 | chr20:35463894 | N_Shelf | C20orf117 | Body |  |
| cg22628146 | 0,11 | 0,17 | -0,060 | 0,000127724 | chr1:45957456 | S_Shore | TESK2 | TSS1500 |  |
| cg03515656 | 0,64 | 0,73 | -0,094 | 0,000137877 | chr16:1600304 | S_Shore | IFT140;  TMEM204 | Body;Body |  |
| cg26632831 | 0,81 | 0,86 | -0,053 | 0,000146298 | chr11:70935863 | OpenSea | SHANK2 | TSS200 |  |
| cg19837587 | 0,03 | 0,08 | -0,052 | 0,000151094 | chr15:51201261 | Island | AP4E1 | Body |  |
| cg18817654 | 0,87 | 0,91 | -0,038 | 0,000161244 | chr15:39485138 | OpenSea |  |  |  |
| cg02259934 | 0,93 | 0,95 | -0,023 | 0,000167901 | chr19:4566484 | Island |  |  |  |
| cg25951430 | 0,32 | 0,52 | -0,198 | 0,000186756 | chr11:57192432 | N_Shore | SLC43A3 | Body |  |
