# Supplementary material for: DNA methylation profiling identifies novel markers of progression in hepatitis B-related chronic liver disease
Source: Clin Epigenetics. 2016 May 5;8:48. doi: 10.1186/s13148-016-0218-1 (PMC4857425; doi:10.1186/s13148-016-0218-1)
Supplement: Additional file 7: Table S4. — Demographic, clinical and laboratory characteristics of the non-progressors and progressors in serial biopsies. (DOCX 18 kb) [file 13148_2016_218_MOESM7_ESM.docx]

**Supplementary Table 4**.

*Demographics, clinical and laboratory characteristics of the non-progressors and progressors in serial biopsies*

|  | Non-progressor  n=6 | Progressor  n=5 | P value |
| --- | --- | --- | --- |
| Gender -male ^a^ | 4 (66,6) | 4 (80) | ns |
| Age (years) ^a^ | 48,8±14,47 | 43,2±4,5 | ns |
| Hbe Ag positive ^a^ | - (0) | 1 (20) | ns |
| Anti Hbe Ab positive ^a^ | 6 (100) | 4 (80) | - |
| HBV DNA ( log IU/ml) ^b^ | 6,7±4,0 | 6,2±4,3 | ns |
| Serum ALT (IU/L) ^b^ | 35,5±10,1 | 46,2±13,9 | ns |
| Serum AST (IU/L) ^b^ | 54,8±30,2 | 62,4±21,1 | ns |
| White blood cell count (x10^9^/L) ^b^ | 6,4±1,5 | 6,3±1,7 | ns |
| Platelet count (x10^9^/L) ^b^ | 181,5±40 | 168±42,7 | ns |
| Hemoglobin (g/L) ^b^ | 13,3±2,1 | 13,4±2,7 | ns |
| Albumin (g/L) ^b^ | 4,3±0,2 | 4,6±0,4 | ns |
| Prothrombin time (s) ^b^ | 11,9±0,6 | 11,4±0,7 | ns |
| Conjugated bilirubin (mg/dL) ^b^ | 0,2±0,1 | 0,3±0,1 | ns |
| Modified histologic activity index ^a^ |  |  |  |
| 0-4 | 3 (50,0) | 3 (60) | ns |
| 5-9 | 3 (50,0) | 2 (40) | ns |
| 10-14 | - | - | - |
| 15-18 | - | - | - |
| Fibrosis  ^a^ |  |  |  |
| 0 | 3 (50,0) | 3 (60,0) | ns |
| 1 | 2 (33,3) | 2 (40,0) | ns |
| 2 | 1 (16,6) | - | - |
| 3 | - | - | - |
| 4 | - | - | - |
| 5 | - | - | - |
| 6 | - | - | - |
